# Dietary Intake Relative to Japanese Reference Values During Late Pregnancy Among Japanese Women Undergoing Scheduled Cesarean Delivery: A Single-Center Cross-Sectional Study

**DOI:** 10.7759/cureus.116748

**Published:** 2026-09-22

**Authors:** Hiroshi Miyabayashi, Noriko Miyazawa, Tatsuo Yamamoto, Mai Q Nguyen, Kinuyo Munakata, Midori Natsume, Yoshitaka Nakamura, Nobuhiko Nagano, Ichiro Morioka

**Affiliations:** 1 Department of Pediatrics, Kasukabe Medical Center, Kasukabe, JPN; 2 Department of Pediatrics and Child Health, Nihon University School of Medicine, Itabashi, JPN; 3 Department of Applied Nutrition, Japan Nutrition University, Sakado, JPN; 4 Department of Obstetrics and Gynecology, Kasukabe Medical Center, Kasukabe, JPN; 5 Wellness Science Labs, Meiji Holdings Co. Ltd, Tokyo, JPN; 6 R&amp;D Division, Meiji Co. Ltd., Tokyo, JPN

**Keywords:** dietary reference intakes, diet history questionnaire, japanese women, micronutrient intake, pregnancy

## Abstract

Background: Maternal nutrition during late pregnancy is crucial for maternal and fetal health. This study descriptively evaluated dietary intake relative to the Dietary Reference Intakes for Japanese (2025 edition) among Japanese women admitted for scheduled cesarean delivery at a single center.

Methods: This single-center cross-sectional study enrolled Japanese women admitted to Kasukabe Medical Center for scheduled cesarean delivery between October 2022 and April 2024. Dietary intake during the preceding month was assessed upon admission using a validated self-administered Diet History Questionnaire. Nutrient intakes were expressed relative to the corresponding values in the Dietary Reference Intakes for Japanese (2025 edition). These reference values were used as descriptive benchmarks and not as diagnostic cutoffs for individual nutritional adequacy. Gestational weight gain was assessed using the Japanese Obstetric Guidelines. The analyses were descriptive. Data on dietary supplement use were not systematically collected.

Results: Of the 58 enrolled women, one (1.7%) was excluded because of incomplete questionnaire data, and 57 (98.3%) were included in the final analysis. The mean energy intake was 1,866 ± 573 kcal/day, and the mean intake relative to the individually estimated energy requirement was 89.6%. The mean intake as a percentage of the reference value was lowest for iron (44.9%), vitamin D (49.3%), and folate (55.8%). The numbers of participants reaching the corresponding reference values were none (0%) for iron, two (3.5%) each for vitamin D and folate, four (7.0%) for vitamin B6, three (5.3%) for magnesium, and four (7.0%) for zinc. The mean sodium intake was 3,961 ± 1,410 mg/day, and only five (8.8%) participants had an intake within the dietary goal. Overall, 32 (56.1%) participants reached at least the lower limit of the recommended gestational weight gain.

Conclusions: In this selected single-center cohort of Japanese women undergoing scheduled cesarean delivery, dietary intakes of several micronutrients were low relative to the Japanese reference values, whereas sodium intake frequently exceeded the dietary goal. These descriptive findings may not be generalizable to all pregnant Japanese women and should not be interpreted as evidence of inadequate total nutrient intake or biochemical nutrient deficiency because dietary supplement use and biochemical nutrient status were not assessed. Larger, more representative multicenter studies that systematically assess supplement use are needed.

## Introduction

Maternal dietary intake during pregnancy is important for maternal and neonatal health. Adequate maternal nutrition supports fetal growth and development and may influence pregnancy outcomes. Recent studies from East Asia have documented low dietary intakes of multiple micronutrients among pregnant women. A large nationwide cohort study in Japan reported low periconceptional intakes of folate, iron, vitamin D, and other essential nutrients, whereas a recent prospective cohort study in Korea reported a high prevalence of low intakes of vitamin D, iron, calcium, and other nutrients during early pregnancy [1,2]. These findings indicate that suboptimal dietary intake of specific micronutrients remains a critical maternal health concern in East Asia.

In Japan, approximately 20% of women in their 20s and 30s are underweight, a prevalence that is more than double that reported in many Western countries [3]. Prepregnancy underweight is associated with impaired fetal growth and increased risks of adverse perinatal outcomes, including low birth weight, small-for-gestational-age birth, and preterm birth [3]. Although the prevalence of low birth weight in Japan has shown a modest decline over the past two decades, further improvement remains an important public health goal [4]. These observations underscore the importance of assessing maternal dietary intake, while findings obtained from selected clinical populations require cautious interpretation.

Dietary history questionnaires are widely used in epidemiological studies to assess habitual dietary intake. In Japan, a self-administered Diet History Questionnaire (DHQ) has been developed and validated as a practical tool for assessing habitual dietary intake in large populations [5,6]. The validity and reliability of both the DHQ and the brief-type DHQ have also been evaluated in Japanese pregnant women, supporting their use for dietary assessment during pregnancy [7]. The DHQ has also been used to assess dietary intake among Japanese pregnant women [8].

The Dietary Reference Intakes for Japanese recommend an additional energy intake of approximately 450 kcal/day during the third trimester, together with appropriate intakes of essential macro- and micronutrients [9]. However, contemporary data describing food and nutrient intake specifically during late pregnancy and comparing estimated dietary intake with the Dietary Reference Intakes for Japanese (2025 edition) remain limited.

In this study, the reference values were used as descriptive benchmarks for dietary intake and not as diagnostic thresholds for individual nutritional deficiency. Accordingly, this single-center cross-sectional study aimed to descriptively characterize dietary and food group intakes during the preceding month among Japanese women admitted for scheduled cesarean delivery in late pregnancy and to compare their estimated dietary intakes with the Dietary Reference Intakes for Japanese (2025 edition). The objective of this study was limited to describing this defined clinical cohort; the study was not intended to estimate dietary intake among all pregnant Japanese women or at other stages of pregnancy.

## Materials and methods

Participants

This single-center cross-sectional study used data from the same cohort as our previous study [10], which examined the association between maternal nutrition and oxidative stress markers during pregnancy. The present analysis addressed a distinct research question and descriptively evaluated dietary intake during late pregnancy relative to the Dietary Reference Intakes for Japanese.

Pregnant Japanese women admitted to the Department of Obstetrics and Gynecology at Kasukabe Medical Center for scheduled cesarean delivery between October 2022 and April 2024 were prospectively enrolled. Enrollment was restricted to scheduled admissions so that the DHQ could be administered at a standardized pre-delivery time point. Accordingly, the study population represents a selected clinical cohort of women undergoing scheduled cesarean delivery at a single institution and was not intended to be representative of all pregnant Japanese women. Because the DHQ assessed dietary intake during the preceding month, the dietary data reflect late pregnancy only and were not used to characterize dietary intake throughout pregnancy.

Women with multiple pregnancies and those of non-Japanese ethnicity were excluded. Participants with incomplete DHQ data were excluded from the final analysis. Clinical and obstetric data included in the analytical dataset were retrospectively extracted from the electronic medical records. The analytical dataset was irreversibly de-identified before the present analysis and could no longer be linked to the source medical records. Information on dietary supplement use was not systematically collected; therefore, the nutrient intake estimates in this study reflect intake from foods rather than total intake from foods and supplements.

This study was approved by the Institutional Review Board of Kasukabe Medical Center (KMC-C2022-014; amendment KMC-C2024-001) and was conducted in accordance with the Declaration of Helsinki. Written informed consent was obtained from all participants during their pregnancies.

Dietary Assessment

Dietary intake during the previous month was assessed on the day of admission for scheduled cesarean delivery using a self-administered DHQ [5-7]. The DHQ is a validated self-administered questionnaire developed in Japan to estimate habitual food and nutrient intake. The participants completed the questionnaire in their hospital rooms before delivery.

Because the Dietary Reference Intakes for Japanese are expressed as absolute daily intake values, estimated absolute nutrient intakes without energy adjustment were used for the primary comparisons. Energy-adjusted values were not used because they cannot be directly compared with absolute dietary reference values. The DHQ has demonstrated acceptable validity and reliability for dietary assessment in Japanese adults and pregnant women and was, therefore, considered an appropriate dietary assessment tool for the present study [5-7].

Estimated daily food group, energy, and nutrient intakes were calculated using the DHQ calculation algorithm based on the Standard Tables of Food Composition in Japan. Maternal energy requirements were estimated as standard body weight (height [m]² × 22) multiplied by 30 kcal/kg/day, with an additional 450 kcal/day for the third trimester [9,11], based on a commonly used clinical estimation method.

Nutrient reference values were based on the Dietary Reference Intakes for Japanese (2025 edition) issued by the Ministry of Health, Labour, and Welfare [9], including additional requirements for late pregnancy, where applicable. Age-specific reference values for pregnant women aged 18-29 and 30-49 years were applied to each participant.

For each nutrient, the reference value was defined as 100%, and individual intake was expressed as a percentage of this value [9]. Because the Dietary Reference Intakes do not specify reference values for most food groups, food group intakes were descriptively summarized. The reference values were used as descriptive benchmarks for comparing dietary intake and were not treated as diagnostic cutoffs for individual nutritional adequacy or deficiency. For nutrients with an adequate intake (AI), intake at or above the reference value was reported descriptively and was not interpreted as confirmation of individual nutrient adequacy.

The participants were categorized according to prepregnancy body mass index (BMI), and the proportion of women whose gestational weight gain reached at least the lower limit of the recommended range was calculated according to the Japanese obstetric guidelines [12].

Statistical analyses

Statistical analyses were performed using R version 4.5.2 (R Foundation for Statistical Computing, Vienna, Austria; https://www.R-project.org/) and EZR version 1.70 (Saitama Medical Center, Jichi Medical University, Saitama, Japan) [13]. Continuous variables were summarized as means ± standard deviations and categorical variables as numbers and percentages. Descriptive statistics were used to summarize nutrient intake, food group intake, and gestational weight gain.

Use of artificial intelligence

During the preparation of this manuscript, ChatGPT (OpenAI) was used to assist with English language editing and to improve readability. The authors have reviewed and edited all AI-assisted output and undertake complete responsibility for the accuracy and content of the manuscript.

## Results

Participants

During the study period, 58 participants were enrolled. One participant (1.7%) was excluded because of incomplete DHQ data, leaving 57 participants (98.3%) for the final analysis. The participant characteristics are summarized in Table 1.

**Table 1:** Participants’ clinical characteristics. Values are presented as mean ± standard deviation (SD) or number (%). Z scores are calculated based on gestational age-specific reference values. BMI, body mass index.

Variable	
N	57
Maternal age, years	34.4 ± 4.7
Prepregnancy weight, kg	56.8 ± 9.0
Prepregnancy BMI, kg/m^2^	22.8 ± 3.6
Maternal weight at delivery, kg	65.9 ± 9.0
Weight gain during pregnancy, kg	9.0 ± 3.7
Primiparous/multiparous, n (%)	18 (31.6%)/39 (68.4%)
Smoking history/smoking during pregnancy, n (%)	12 (21.1%)/0 (0%)
History of alcohol consumption/alcohol use during pregnancy, n (%)	47 (82.5%)/7 (12.3%)
Gestational age, weeks	37.9 ± 0.8
Birth weight, g	2,877 ± 329
Birth weight, Z score	0.20 ± 0.84
Head circumference, cm	33.3 ± 1.1
Head circumference, Z score	0.28 ± 0.79
Male/female, n (%)	28 (49.1%)/29 (50.9%)

The mean maternal age was 34.4 ± 4.7 (range, 22-43) years, and the mean prepregnancy BMI was 22.8 ± 3.6 kg/m². Eighteen (31.6%) women were primiparous, and 39 (68.4%) were multiparous.

Nutrient intake relative to reference values

Table 2 and Figure 1 show nutrient intakes during late pregnancy relative to the Dietary Reference Intakes for Japanese (2025 edition). The mean energy intake was 1,866 ± 573 kcal/day, the mean individually estimated energy requirement was 2,097 ± 114 kcal/day, and the mean individual energy intake-to-requirement ratio was 89.6 ± 30.6%.

**Table 2:** Nutrient intakes relative to reference values during late pregnancy. Values are presented as mean ± standard deviation (SD), unless otherwise indicated. Mean intake relative to the reference value was calculated as individual intake divided by the corresponding reference value × 100. Vitamin A values are expressed as RAE, vitamin E values as α-tocopherol equivalents, and niacin values as NE. Reference values were based on the Dietary Reference Intakes for Japanese (2025 edition), including additional requirements for late pregnancy where applicable. For nutrients with age-specific reference values, values are presented for women aged 18–29 years/30–49 years, respectively. Reference values were used as descriptive benchmarks and not as diagnostic cutoffs for individual nutritional adequacy or deficiency. The numbers and percentages of participants reaching the reference values are presented descriptively and should not be interpreted as the prevalence of individual nutritional adequacy. In particular, for nutrients with an AI, an intake at or above the AI does not confirm individual nutrient adequacy. AI, adequate intake; DG, tentative dietary goal for preventing lifestyle-related diseases; EER, estimated energy requirement; NE, niacin equivalents; RAE, retinol activity equivalents; RDA, recommended dietary allowance; SD, standard deviation. ^*^Number and percentage of participants whose sodium intake was below the dietary goal (<2,559 mg/day). For sodium, the reference value represents the upper limit of the dietary goal; therefore, a lower intake indicates that the dietary goal was met.

Variable	Intake, mean ± SD	Reference value	Reference type	Mean intake, % of reference value	Participants reaching reference value, n (%)
Energy intake, kcal/day	1,866 ± 573	2,097 ± 114	EER	89.6 ± 30.6	10 (17.5)
Vitamin A, µg RAE/day	479.5 ± 267.5	730/780	RDA	62.2 ± 34.9	8 (14.0)
Vitamin D, µg/day	4.4 ± 2.1	9.0	AI	49.3 ± 22.8	2 (3.5)
Vitamin B1, mg/day	0.8 ± 0.3	1.0/1.1	RDA	77.6 ± 26.1	11 (19.3)
Vitamin B2, mg/day	1.4 ± 0.5	1.5	RDA	90.3 ± 30.7	18 (31.6)
Niacin, mg NE/day	12.6 ± 4.0	11/12	RDA	106.4 ± 35.0	28 (49.1)
Vitamin B6, mg/day	0.9 ± 0.3	1.4	RDA	67.2 ± 22.9	4 (7.0)
Vitamin B12, µg/day	4.1 ± 2.1	4.0	AI	103.5 ± 53.2	27 (47.4)
Folate, µg/day	267.8 ± 91.8	480	RDA	55.8 ± 19.1	2 (3.5)
Pantothenic acid, mg/day	5.7 ± 1.7	5.0	AI	114.3 ± 35.0	37 (64.9)
Vitamin C, mg/day	90.6 ± 47.3	110	RDA	82.3 ± 43.0	13 (22.8)
Vitamin E, mg/day	7.8 ± 3.9	5.5	AI	141.5 ± 70.1	45 (78.9)
Sodium, mg/day	3,961 ± 1,410	<2,559	DG	154.8 ± 55.1	5 (8.8)*
Potassium, mg/day	2,034 ± 645	2,000	AI	101.7 ± 32.2	22 (38.6)
Calcium, mg/day	545.1 ± 214.8	650	RDA	83.9 ± 33.0	14 (24.6)
Magnesium, mg/day	215.5 ± 65.8	320/330	RDA	65.7 ± 20.2	3 (5.3)
Phosphorus, mg/day	931 ± 281	800	AI	116.4 ± 35.1	35 (61.4)
Iron, mg/day	6.5 ± 2.1	14.5	RDA	44.9 ± 14.6	0 (0.0)
Zinc, mg/day	7.2 ± 1.9	9.5/10	RDA	72.6 ± 19.4	4 (7.0)
Copper, mg/day	1.0 ± 0.3	0.8	RDA	123.8 ± 35.0	44 (77.2)
Manganese, mg/day	3.8 ± 1.6	3.0	AI	125.7 ± 53.0	35 (61.4)

**Figure 1:**
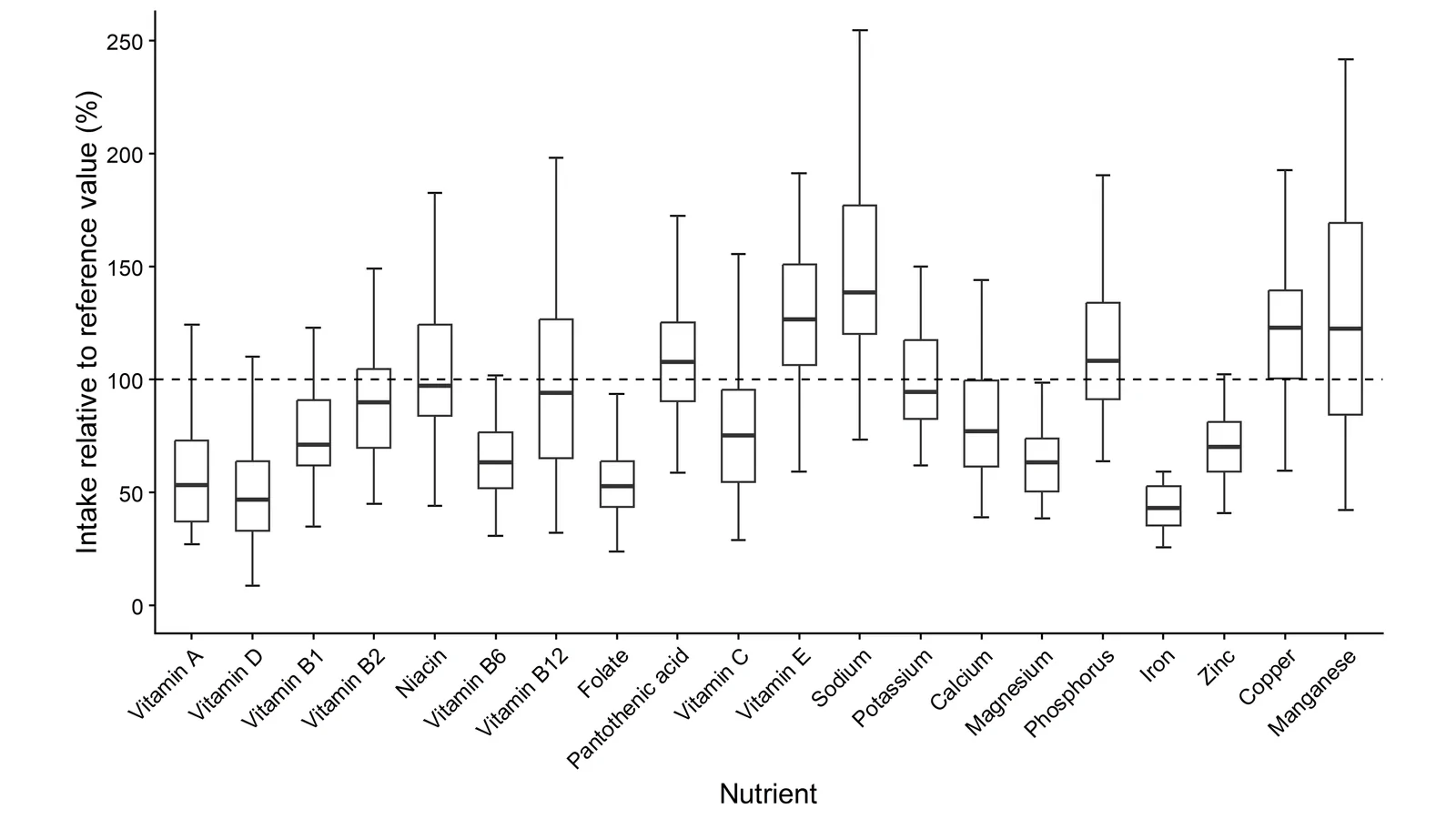
Distribution of nutrient intake relative to reference values (%) during late pregnancy. Nutrient intake was expressed as a percentage of the corresponding reference value in the Dietary Reference Intakes for Japanese (2025 edition), with age-specific reference values applied where appropriate. The dashed line represents 100% of the reference value. For sodium, values >100% indicate intake exceeding the dietary goal. Outlying points are not displayed for clarity; all observations were retained in the analyses. Reference values were used as descriptive benchmarks and should not be interpreted as diagnostic cutoffs for individual nutritional adequacy.

Several micronutrients showed low dietary intake relative to the reference values. Iron had the lowest mean intake relative to the reference value (44.9%), followed by vitamin D (49.3%) and folate (55.8%). The numbers of participants reaching the corresponding reference values were none (0%) for iron, two (3.5%) each for vitamin D and folate, four (7.0%) for vitamin B6, three (5.3%) for magnesium, and four (7.0%) for zinc. The mean sodium intake was 3,961 ± 1,410 mg/day, exceeding the dietary goal of <2,559 mg/day, and only five (8.8%) participants had an intake within the dietary goal. The mean potassium intake corresponded to 101.7% of the AI, with 22 (38.6%) participants having an intake at or above this value. The mean phosphorus intake corresponded to 116.4% of the AI, with 35 (61.4%) participants having an intake at or above this value. Because AI is not a cutoff for determining individual nutritional adequacy, these proportions are presented descriptively and should not be interpreted as the prevalence of individual nutrient adequacy.

Food group intake

The food group intakes are summarized in Table 3. The mean intakes of vegetables, fruits, and seafood were 174 ± 91, 110.3 ± 116.2, and 33.5 ± 18.3 g/day, respectively.

**Table 3:** Food group intakes. Values are presented as the means ± standard deviations.

Variable	Intake, mean ± standard deviation
Nuts and seeds, g/day	1.2 ± 2.6
Potatoes, g/day	24.5 ± 15.6
Beans, g/day	46.9 ± 38.6
Fruits, g/day	110.3 ± 116.2
Total vegetables, g/day	174 ± 91
of which: Green and yellow vegetables, g/day	82.6 ± 51.0
of which: Other vegetables, g/day	91.8 ± 59.3
Mushrooms, g/day	10.8 ± 8.9
Seaweed, g/day	8.9 ± 14.2
Seafood, g/day	33.5 ± 18.3
Meat, g/day	76.2 ± 37.1
Eggs, g/day	24.5 ± 21.0
Milk, g/day	175.2 ± 140.9

Gestational weight gain

The gestational weight gain according to the prepregnancy BMI category is presented in Table 4. Overall, 32 (56.1%) participants reached at least the lower limit of the recommended gestational weight gain. The corresponding numbers and proportions were 1 (33.3%), 23 (52.3%), 5 (71.4%), and 3 (100%) among women with prepregnancy BMI < 18.5, 18.5-24.9, 25.0-29.9, and ≥ 30 kg/m², respectively.

**Table 4:** Gestational weight gain according to prepregnancy BMI categories. Values are presented as means ± standard deviations or numbers (%). The participants were classified as having reached the recommended minimum gestational weight gain if their weight gain was equal to or greater than the lower limit specified in the Japanese obstetric guidelines (2020). BMI: body mass index.

Prepregnancy BMI	N	Gestational weight gain, kg	Recommended weight gain, kg	Reached lower limit of recommended weight gain, n (%)
<18.5 kg/m²	3	10.7 ± 1.5	12–15	1 (33.3)
18.5–24.9 kg/m²	44	9.2 ± 4.0	10–13	23 (52.3)
25.0–29.9 kg/m²	7	7.7 ± 2.4	7–10	5 (71.4)
≥30 kg/m²	3	8.0 ± 3.6	5 (individualized guideline)	3 (100)

## Discussion

In this selected single-center cohort of Japanese women admitted for scheduled cesarean delivery during late pregnancy, dietary intakes of several micronutrients were low relative to the Dietary Reference Intakes for Japanese, despite the mean energy intake being close to the estimated requirement. The lowest mean intakes relative to the reference values were observed for iron (44.9%), vitamin D (49.3%), and folate (55.8%). The number of participants reaching the corresponding reference values was none (0%) for iron, two (3.5%) each for vitamin D and folate, four (7.0%) for vitamin B6, three (5.3%) for magnesium, and four (7.0%) for zinc. In contrast, only five (8.8%) participants had sodium intake within the dietary goal, whereas phosphorus intake was at or above the AI in 35 (61.4%) participants. These findings describe dietary intake in this selected clinical cohort and should not be generalized to all pregnant Japanese women. Furthermore, the reference values were used as descriptive benchmarks and should not be interpreted as diagnostic cutoffs for individual nutritional adequacy or deficiency.

The pattern observed in this selected cohort is broadly consistent with recent reports of low dietary intakes of several micronutrients among pregnant women in Japan and Korea. Low dietary intakes of iron, vitamin D, and folate have been reported in Japan, and a recent Korean cohort reported high prevalence of low intakes of vitamin D, iron, calcium, and other nutrients during early pregnancy [1,2]. Although direct comparisons are limited by differences in study populations and gestational periods, these reports suggest that low dietary intakes of several micronutrients may occur across different East Asian pregnant populations.

Among the nutrients evaluated, iron had the lowest mean intake relative to the reference value. Because iron requirements substantially increase during pregnancy owing to the expansion of maternal blood volume and fetal iron demands, achieving the reference intake through diet alone may be difficult [9]. This may partly explain why dietary iron intake was substantially below the reference value in this cohort.

Vitamin D and folate also showed markedly low intakes relative to the reference values. Folate is essential for fetal growth and neural tube development, whereas vitamin D plays an important role in maternal bone metabolism and fetal skeletal development [9]. The low dietary intakes observed in this cohort are consistent with a previous nationwide Japanese report [1], although the present findings should not be generalized beyond this selected population.

At the group level, the mean energy intake was close to but below the estimated requirement (89.6%), and 32 (56.1%) participants reached at least the lower limit of the recommended gestational weight gain. Nevertheless, energy intake close to the estimated requirement did not correspond to intakes reaching the reference values for several micronutrients, suggesting that the nutrient balance, rather than caloric intake alone, was suboptimal.

The pattern observed in this cohort is also consistent with previous Japanese studies and reviews describing suboptimal nutritional intake and malnutrition concerns among women before and during pregnancy [1,8,14]. Although the Dietary Reference Intakes for Japanese specify additional energy and nutrient requirements during pregnancy [9], the reference values for several micronutrients were reached by only a small proportion of participants in this cohort. This finding highlights the potential importance of considering nutrient density and micronutrient balance in addition to energy intake. Because this study assessed dietary intake rather than biochemical nutrient status, the findings should be interpreted as low dietary intake relative to reference values rather than confirmed nutrient deficiency.

The low dietary intakes relative to the reference values observed in this study may partly reflect habitual food choices. In a recent cross-sectional study of Japanese adults, participants in their 20s reported lower intake frequencies of fish, soybeans, dairy products, seaweed, fruits, potatoes, and, in some comparisons, vegetables than older participants [15]. In this cohort, the mean intakes of vegetables, fruits, and seafood were 174, 110.3, and 33.5 g/day, respectively. These food groups are sources of several micronutrients for which dietary intake was low relative to the reference values; therefore, the observed food group intake pattern may have contributed to these findings. However, because associations between food group intake and nutrient intake relative to reference values were not directly examined, this interpretation should be considered exploratory. Appropriate weight management also remains important because gestational weight gain below or above the recommended range is associated with distinct adverse maternal and neonatal outcomes [12,16].

This study also identified high sodium intake, whereas mean potassium intake was close to the AI in Japan. The mean sodium intake substantially exceeded the dietary goal, and only five (8.8%) participants had an intake within the dietary goal. The mean potassium intake was 101.7% of the AI, and 22 (38.6%) participants had an intake at or above this value. However, because the AI is not a cutoff for classifying individual nutrient inadequacy, this percentage should be cautiously interpreted. The combination of high sodium intake and mean potassium intake near the AI may be relevant to blood pressure regulation and highlights the importance of considering the sodium-potassium balance in prenatal dietary counseling [9,17].

Phosphorus intake was at or above the AI in 35 (61.4%) participants. However, this finding should not be interpreted as excessive intake solely based on exceeding AI, because AI is not an upper safety threshold. The Dietary Reference Intakes for Japanese set an AI of 800 mg/day and a tolerable upper intake level of 3,000 mg/day for adults [9]. Because the dietary sources of phosphorus were not directly examined, the contributions of individual food groups or phosphate-containing food additives could not be determined.

This study has certain limitations. First, this was a single-center study with a relatively small sample size, which may have limited the generalizability and stability of the findings.

Second, the study population was restricted to women admitted for scheduled cesarean delivery and therefore represents a highly selected clinical cohort rather than the general population of pregnant Japanese women. Although this sampling strategy enabled dietary assessment at a comparable stage of late pregnancy and under standardized pre-delivery conditions, women undergoing scheduled cesarean delivery may differ from the broader pregnant population in obstetric characteristics, health behaviors, underlying conditions, and dietary patterns. Detailed indications for scheduled cesarean delivery were not available in the irreversibly de-identified analytical dataset; therefore, this study could not assess whether specific obstetric conditions or condition-related dietary counseling influenced dietary intake. Consequently, substantial selection bias cannot be excluded, and the present findings should not be generalized to all pregnant Japanese women.

Third, dietary intake was self-reported at a single time point using a self-administered DHQ rather than being assessed through direct or repeated dietary measurements. Therefore, recall errors, misreporting, and day-to-day or seasonal variations may have affected the estimated intake values.

Fourth, energy requirements were estimated using a standardized clinical formula without an individual assessment of physical activity. Consequently, energy intake relative to the estimated requirement may have been imprecisely estimated.

Fifth, information regarding dietary supplement use was not systematically collected. Because supplements may substantially contribute to the intake of folate, iron, vitamin D, and other nutrients during pregnancy, the total nutrient intake may have been underestimated. Accordingly, the present findings reflect nutrient intake from the diet rather than total intake and should not be interpreted as evidence of biochemical nutrient deficiency.

Finally, this was a descriptive observational study that was not designed or powered to examine the association between dietary intake relative to reference values and gestational weight gain, maternal complications, or neonatal outcomes. Therefore, no causal inferences can be drawn from the present findings.

## Conclusions

In this selected single-center cohort of Japanese women undergoing scheduled cesarean delivery during late pregnancy, dietary intakes of several micronutrients, particularly iron, vitamin D, and folate, were low relative to the Dietary Reference Intakes for Japanese, whereas sodium intake frequently exceeded the dietary goal. These descriptive findings suggest that energy intake close to the estimated requirement does not necessarily correspond to dietary intakes reaching reference values for all micronutrients. Because of the selected study population, small sample size, and lack of systematic information on dietary supplement use, the findings should not be generalized to all pregnant Japanese women. Larger, multicenter studies involving more representative pregnant populations and systematically assessing supplement use are required.
